# Effects of neoadjuvant immunotherapy on hearing in patients with head and neck squamous cell carcinoma

**DOI:** 10.1038/s41598-025-13706-9

**Published:** 2025-07-29

**Authors:** Bryce Hambach, Anhelina Bilokon, John Lee, L. Noelle Allemang, Jennifer Chisholm, Chris K. Zalewski, Julie Christensen, Carmen C. Brewer, James L. Gulley, Clint T. Allen, Lisa L. Cunningham, Katharine A. Fernandez

**Affiliations:** 1 Laboratory of Hearing Biology and Therapeutics, National Institute on Deafness and Other Communication Disorders (NIDCD), NIH, Bethesda, MD USA; 2 Sidney Kimmel Medical College, Thomas Jefferson University, Philadelphia, PA USA; 3 Auditory and Vestibular Clinical Research Section (AVCRS), National Institute on Deafness and Other Communication Disorders (NIDCD), NIH, Bethesda, MD USA; 4 Center for Immuno-Oncology, Center for Cancer Research, National Cancer Institute (NCI), NIH, Bethesda, MD USA; 5 Surgical Oncology Program, Center for Cancer Research, National Cancer Institute (NCI), NIH, Bethesda, MD USA

**Keywords:** Immune checkpoint blockade (ICB), Neoadjuvant therapy, Hearing loss, Head and neck cancer, Ototoxicity, Long-term monitoring, Head and neck cancer, Cancer, Oncology

## Abstract

Immune checkpoint blockade (ICB) is a commonly used treatment modality for cancer with a growing list of oncologic indications. Ototoxicity is a potential immune-related adverse event of ICB treatment, but the risk of hearing loss after ICB remains unknown. This retrospective chart review sought to identify individuals who received ICB and had available audiometric data before and after treatment in order to identify clinically meaningful changes in hearing. This single center, institutional chart review examined hearing function data in patients who underwent audiometry before and after ICB treatment. Measures included pure tone thresholds, distortion product otoacoustic emissions (DPOAEs), tympanometry, and word recognition scores. Ototoxicity was assessed using the American Speech-Language-Hearing-Association (ASHA) and Common Terminology Criteria for Adverse Events (CTCAEv5.0) guidelines where applicable. We identified twelve individuals with newly diagnosed, advanced-stage Head and Neck Squamous Cell Carcinoma (HNSCC) who received neoadjuvant ICB on a clinical trial. Eleven of twelve (92%) patients (8 male, 4 female, age 41–79 years) did not meet criteria for hearing threshold changes. One patient demonstrated a 10 dB shift at two consecutive frequencies in one ear, meeting the minimum ASHA criteria for significant change. Speech audiometry and tympanometry identified no differences in speech understanding or middle ear function pre-and post-treatment. Variations in DPOAE amplitudes were noted, potentially indicating early outer hair cell damage despite stable audiometric thresholds. In this small data set of newly diagnosed advanced-stage HNSCC, there appears to be a low incidence of significant hearing changes following neoadjuvant ICB treatment. Larger, prospective studies with long-term follow-up are needed to evaluate late-onset ototoxicity. Cancer survivors treated with neoadjuvant immune checkpoint blockade for advanced-stage head and neck squamous cell carcinoma (HNSCC) appear to have a low risk of acute hearing loss based on this study. While significant changes in hearing thresholds were not observed, subtle alterations in cochlear outer hair cell function were detected, suggesting the importance of long-term monitoring to capture potential delayed effects on hearing. This insight helps reassure patients about the immediate ototoxic risks while highlighting the need for continued vigilance in hearing assessment during follow-up.

## Background

 Head and neck cancer remains a significant global health challenge and a cause of morbidity and mortality despite advances in standard anti-cancer treatments such as surgery, chemotherapy, and radiation. The risk of disease relapse remains high for patients with advanced-stage head and neck squamous cell carcinoma (HNSCC)^1,2^. Immune checkpoint blockade (ICB) immunotherapy targeting the programmed cell death protein-1 (PD-1)/PD-1 ligand (PD-L1) axis with or without chemotherapy is now approved as first-line treatment for patients with relapsed disease; however, only about 1/3 of patients demonstrate objective, measurable responses in this clinical setting^3^. Reports from clinical trials of neoadjuvant ICB have shown promising results including reduced disease relapse and improved recurrence-free survival when administered in the perioperative setting for resectable HNSCC^4,5^. Keynote-689 is an ongoing phase 3 study randomizing individuals to neoadjuvant ICB (pembrolizumab) in the perioperative setting plus standard adjuvant therapy vs. standard adjuvant therapy alone for resectable HNSCC (NCT03765918).

Given the growing role of ICB in cancer treatment, it is important to understand the risks associated with neoadjuvant ICB treatment. ICB therapy for relapsed cancer often requires prolonged treatment duration, which is associated with various toxicities and immune-related adverse events that can impact any organ system^6^. Although it is known that some anti-cancer therapies are ototoxic—such as cisplatin, which causes permanent hearing loss in 23–50% of patients^7^ - the risk of ototoxicity resulting from ICB treatment is not well understood. There are case reports of hearing loss following ICB treatment of patients with relapsed cancer^8–10^and engineered cell therapy can cause ototoxicity due to on-target but off-tumor immune damage to the inner ear^11^. Additionally, hearing loss has been observed in pre-clinical studies of rodents treated with ICB^12^.

Despite these pre-clinical and clinical data demonstrating that immunotherapy may carry the risk of ototoxicity, no reports of clinical measures of hearing function before and after ICB treatment exist. Here we report data from a retrospective analysis where twelve patients with newly diagnosed advanced-stage HNSCC who received neoadjuvant ICB on a clinical trial and had available audiologic assessment data were identified.

## Methods

### Study design and patients

We conducted a single-site, institutional chart review to identify adults who had received ICB and also underwent audiologic assessments within 1 month prior to ICB treatment initiation and after receiving ICB treatment. We sought to review audiological evaluations that included pure tone audiometry, speech audiometry, tympanometry, and distortion product otoacoustic emission (DPOAE) testing. All identified audiograms were from individuals that were treated and had audiometric testing performed on the NIH Clinical Center IRB-approved clinical trial NCT04247282 as previously reported^13^. All patients provided verbal and written informed consent to be treated on this study, and the study was conducted in accordance with the Declaration of Helsinki. Approval for retrospective review of audiometric testing was granted by the National Institutes of Health Institutional Review Board (04/16/24, No. IRB01985).

### Auditory tests

We identified individuals who had pure tone audiometric hearing thresholds (lowest level in dB HL at which a response was obtained for at least 50% of stimulus presentations) determined using a GSI AudioStar Pro clinical audiometer (Grason-Stadler, Eden Prairie, MN), with Radioear IP30 insert earphones (Radioear, Middelfart, Denmark) for frequencies 0.25, 0.5, 1, 2, 3, 4, 6, and 8 kHz. Extended high-frequency pure tone thresholds (9, 10, 11.5, 12.5, 14, 16, 18, 20 kHz) were obtained in a subset of the individuals we identified using Radioear DD450 circum-aural headphones.

Speech audiometry is used for clinical assessment of the reception and recognition of speech. A speech reception threshold is first determined in each ear using a 2 dB step size resolution to quantify an individual’s hearing threshold level for speech and serve as a validity check for the pure tone audiogram. Individuals who had word recognition scores (WRS) assessed underwent testing in each ear using recorded monosyllabic NU-6-word lists of 25 words, presented 25 dB above the 2 kHz threshold in dB HL.

Tympanometry is an objective standard clinical measure of middle ear function that is used to evaluate middle ear system mobility and pressure. Identified individuals underwent tympanometry assessment using a GSI Tympstar Pro Diagnostic Middle Ear Analyzer (Grason-Stadler, Eden Prairie, MN). The probe delivered a 226 Hz tone across an applied pressure change from + 200 to −400 daPa while recording changes in acoustic admittance.

DPOAEs are low-level sounds emitted from the cochlea in response to two pure tones closely related in frequency. DPOAEs are standard clinical measures that reflect the integrity and function of the cochlear outer hair cells. Changes in DPOAE amplitudes can be a sensitive marker for early ototoxicity before changes in audiometric thresholds are observed^14,15^. In a subset of identified individuals, DPOAEs were obtained and measured in quarter octave decrements from 842 to 9509 Hz by presenting tone pairs with a frequency (f) ratio of 1.22 and levels set at 65 and 55 dB SPL using ILO V6 Research OAE system (Otodynamics, Hatfield UK). A minimum of two sweeps across the test frequency range are collected for each ear.

### Data analyses for evidence of ototoxicity

Pure tone hearing thresholds for audiometric frequencies ranging from 0.25 to 20 kHz were compiled during chart review. Air conduction threshold shifts were then calculated as the difference in auditory thresholds between the pre-and post-ICB hearing tests at each frequency. During analysis, no data points were calculated or replaced in the event of missing test points. When there was no response at the highest output level of the audiometer, a value of + 5 dB was added to the maximum output level of the audiometer and recorded as the patient’s threshold. Based on the average air conduction hearing thresholds of 0.5, 1, 2, and 4 kHz, hearing loss was classified according to the World Health Organization (2021) as normal < 20 dB HL, mild 20–34 dB HL, moderate 35–49 dB HL, and moderately severe 50–64 dB HL^16^.

National Cancer Institute Common Terminology Criteria for Adverse Events (CTCAEv5.0) were applied to threshold shifts to identify and grade clinically meaningful changes in hearing^17^. CTCAEv5.0 incorporates threshold shift data from 1 to 8 kHz and therefore would best describe a functional impact of ICB treatment on speech-related frequencies (Table 1). American Speech Language Hearing Association (1994) criteria were also applied to threshold shifts between 1 and 12.5 kHz to identify significant changes in hearing thresholds (Table 1)^18^. ASHA criteria are often more sensitive to early signs of ototoxicity due to their inclusion of higher frequencies relative to the conventional audiogram. Ototoxic changes to audiometric thresholds at these higher frequencies can often be detected before damage to regions of the cochlea that correspond with speech.

**Table 1:** Ototoxicity grading scales.

Scale	Ototoxicity Classification Parameters
American Speech-Language- Hearing Association (ASHA) ^18^ – Includes all frequencies on the behavioral audiogram	10-dB change from baseline at 2 consecutive freq., 20-dB change at 1 freq., or loss of response where one was previously obtained.
National Cancer Institute Common Terminology Criteria for Adverse Events (CTCAE) v5 ^17^ – Based on responses to 1, 2, 3, 4, 6, and 8 kHz on the behavioral audiogram	Grade 1: Threshold shift of 15–25 dB averaged at 2 contiguous test frequencies in at least one ear
	Grade 2: Threshold shift of > 25 dB averaged at 2 contiguous test frequencies in at least one ear
	Grade 3: Threshold shift of > 25 dB averaged at 3 contiguous test frequencies in at least one ear; therapeutic intervention indicated
	Grade 4: Adults: Decrease in hearing to profound bilateral loss (absolute threshold > 80 dB HL at 2 kHz and above); non-serviceable hearing

DPOAEs were evaluated using several criteria. DPOAEs that were not tested at both pre-and post-treatment time points were excluded. Changes in the DPOAE response were only assessed, per patient, when the pre-treatment DPOAE amplitude at 2f1-f2 was greater than or equal to two standard deviations above the noise floor. Amplitude shifts for each frequency pair were then calculated as the difference between the pre-and post-treatment amplitude. Currently, there are no widely adopted clinical standards for interpreting changes in DPOAE amplitude. Therefore, reference limits derived from a meta-analysis of 10 DPOAE test-retest studies were applied to our dataset^19^. These reference limits take into account differences in probe fit and in-ear calibration variances. If a patient’s DPOAE amplitude shifts exceeded the suggested test-retest reliability limits at two consecutive frequencies, the change was considered to be related to ototoxicity.

### Outcomes & statistical analyses

The primary objective of this study was to determine the incidence of clinically meaningful changes in hearing, as defined by CTCAEv5.0 and ASHA, after exposure to ICB^17,18^. Data were analyzed using GraphPad Prism software (version 10.3.0). Descriptive statistics were used to evaluate threshold and DPOAE data. Wilcoxon matched-pairs signed rank tests were utilized to compare pre-and post-treatment word recognition scores. The level of significance was set at $\:\alpha\:$<0.05.

## Results

### Patient characteristics

We identified 12 individuals with available audiologic assessment data pre- and post-ICB treatment. All identified individuals were patients with newly-diagnosed, previously-untreated, advanced stage (stage III/IV) squamous cell carcinoma of the oral cavity unrelated to human papillomavirus treated with two doses of bintrafusp alfa, 1,200 mg intravenously every two weeks for two doses, in the neoadjuvant setting prior to definitive surgical resection on clinical trial NCT04247282. Bintrafusp alfa is a bifunctional fusion protein composed of the extracellular domain of the human TGF-β receptor II (TGF-βRII or TGF-β “trap”) fused via a flexible linker to the C-terminus of each heavy chain of an IgG1 antibody blocking programmed death ligand 1 (anti–PD-L1). Oncologic results describing a range of pathologic responses from this clinical trial have been reported previously^13^. The mean age was 57.9 years (Range: 41–79 years), and 8 (67%) patients were male. Data from auditory profiling performed before and after bintrafusp alfa treatment but prior to any surgery or any adjuvant treatment were identified, allowing us to assess the impact of the neoadjuvant immunotherapy on audiometric function without the influence of other treatments.

### Auditory profiling

**Fig. 1:**
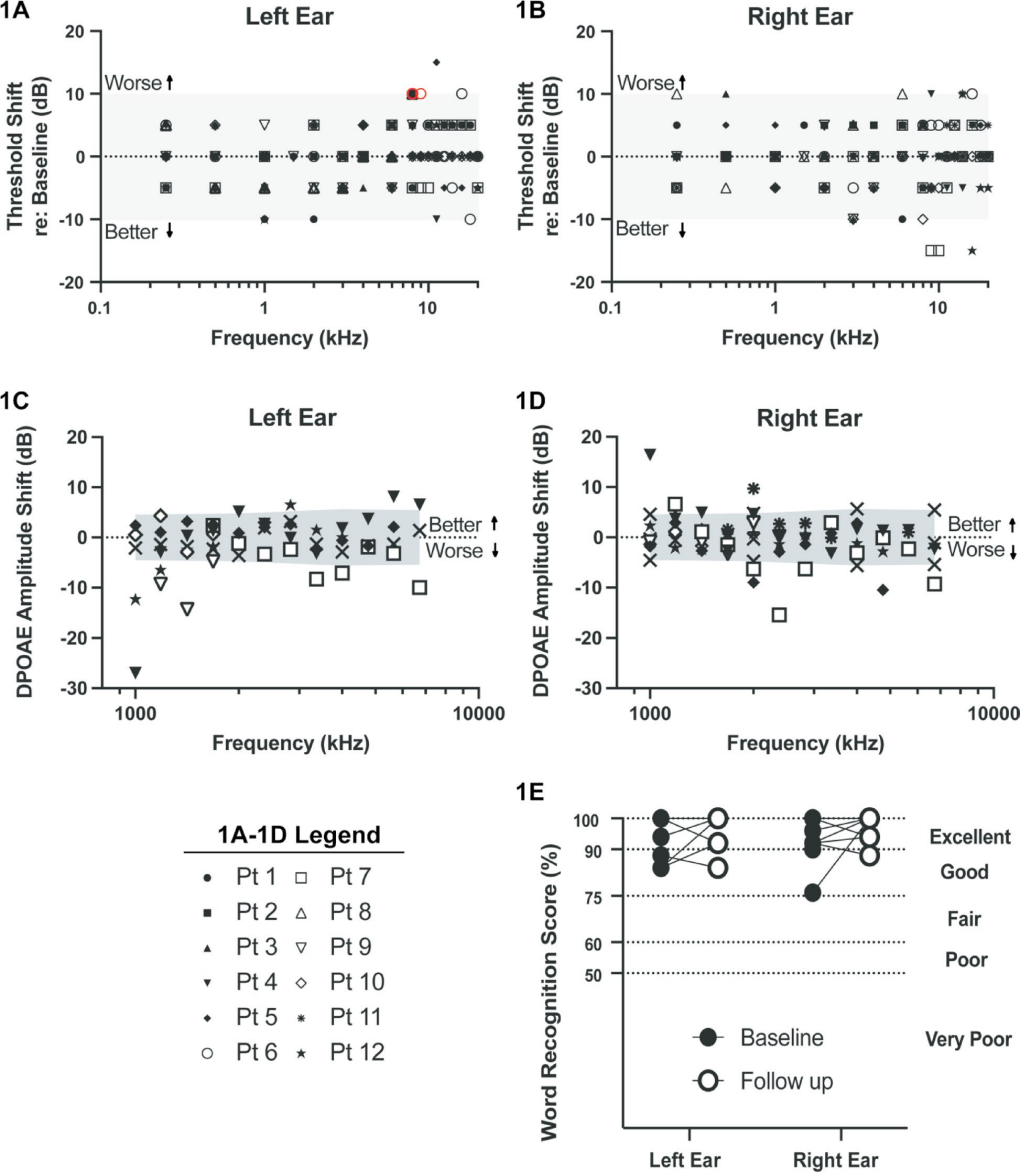
**a & b**: ICB resulted in minimal changes in audiometric hearing sensitivity**.** Data represented are individual behavioral audiogram threshold shifts comparing responses pre- and post-treatment with bintrafusp alfa. Threshold shifts are based on air conduction pure tone thresholds. Grey-shaded regions denote the refence limits of expected re-test reliability. Red symbols represent the left ear thresholds of participant 6 that met the minimum ASHA criteria for a change in hearing function. *N* = 12. **c & d**: DPOAE data suggest that some patients had changes in cochlear outer hair cell function. Grey-shaded regions denote the refence limits of expected re-test reliability for DPOAE amplitudes^19^. Shifts were calculated relative to pre-treatment DPOAE amplitudes at 2f1-f2 where amplitudes exceeded 2SD of the noise floor. *N* = 8. 1e: No ICB-related changes in word recognition scores were observed. Word recognition scores in quiet were collected at baseline for right (mean +/- SD, 93.3% +/- 7.28) and left (mean +/- SD, 92.2% +/- 7.36) ears. At follow-up, means were again collected for right (mean +/- SD, 97.0% +/- 4.54) and left (mean +/- SD, 96.0% +/- 6.53) ears. Performance on WRS tests was categorized from Excellent (90%) to Very Poor (< 50%) according to Silman, 1997 ^20^. Data represent maximum scores in the event that multiple presentation levels were used. *N* = 8.

 Nine patients had normal hearing (*n* = 6) or mild hearing loss (*n* = 3) bilaterally prior to treatment (Table 2). One patient (Pt. 10) had a history of unilateral noise trauma prior to the study and presented with a unilateral moderately severe hearing loss. Post-treatment assessments revealed no change in hearing function for 11 of 12 (92%) patients. However, one patient (Pt. 2) was reclassified from bilateral mild hearing loss pre-treatment to mild hearing loss (right ear) and normal hearing (left ear) post-treatment. This apparent improvement in hearing may be attributable to either test-retest reliability or the tumor size reduction and is likely not related a direct effect of bintrafusp alfa. Based on calculated threshold shift data (Fig. 1A and B), no patient met CTCAE criteria for ICB-related hearing loss. One patient (Pt. 6) met the minimum ASHA criteria (Fig. 1B, shown in red) due to 10 dB threshold shifts at two consecutive frequencies (8 and 9 kHz) in their left ear only.

**Table 2:** Auditory Profiles.

Patient ID	Left Ear						Right Ear							
	Hearing Status^#^		Word Recognition		Middle Ear Status*		Hearing Status^#^			Word Recognition		Middle Ear Status*		
	Baseline	Post-tx	Baseline	Post-tx	Baseline	Post-tx		Baseline	Post-tx	Baseline	Post-tx		Baseline	Post-tx
1	Moderate	Moderate	DNT	DNT	Normal	n/a		Mild	Mild	DNT	DNT		Normal	n/a
2	Mild	Mild	DNT	DNT	Flat (immobile)	Flat (immobile)		Mild	Normal	DNT	DNT		Hypo-mobility	Hypo-mobility
3	Mild	Mild	84	100	Normal	Normal		Mild	Mild	92	94		Negative pressure	Negative pressure
4	Normal	Normal	100	100	Normal	Normal		Normal	Normal	100	100		Normal	Normal
5	Normal	Normal	DNT	DNT	Normal	Normal		Normal	Normal	DNT	DNT		Normal	Normal
6	Normal	Normal	DNT	DNT	Normal	n/a		Normal	Normal	DNT	DNT		Normal	n/a
7	Normal	Normal	100	92	Normal	Normal		Normal	Normal	92	88		Normal	Normal
8	Moderate	Moderate	84	92	Hypo-mobility	Normal		Mild	Mild	76	100		Normal	Normal
9	Mild	Mild	100	100	Normal	Normal		Mild	Mild	100	100		Normal	Normal
10	Moderately-Severe	Moderately-Severe	88	84	Hypo-mobility	Hypo-mobility		Normal	Normal	100	94		Hypo-mobility	Hypo-mobility
11	Normal	Normal	94	100	Normal	Normal		Normal	Normal	92	100		Normal	Normal
12	Normal	Normal	100	100	Normal	Normal		Normal	Normal	96	100		Hyper-mobility	Hyper-mobility

^#^ Based on WHO World Report on Hearing 2021^16^, degree of hearing loss per ear was based on the air conduction (AC) pure tone average (PTA) of 0.5, 1, 2, and 4 kHz: Normal (< 20 dB HL), Mild (≥ 20, < 35 dB HL), Moderate (≥ 35, < 50 dB HL), Moderately Severe (≥ 50, < 65 dB HL). * Based on Margolis and Heller, 1987^25^. Word Recognition values represent % correct using NU-6 word lists. DNT: Did not test.

Tympanometry results were available in 10 subjects pre- and post-treatment (Table 2). Six patients had symmetrical middle ear status, of which were five considered to have bilateral normal function, and one had bilateral hypomobility. Four patients had asymmetrical middle ear status, where one ear demonstrated normal function, and the other either hypermobility (*n* = 1), hypomobility (*n* = 2), or with negative pressure (*n* = 1). Following treatment, middle ear status across nine patients remained consistent with pre-treatment results. One patient with hypomobility pre-treatment (Pt. 8) demonstrated normal middle ear function during the post-treatment assessment. Thus, we did not identify significant changes in middle ear function post-ICB therapy.

### Distortion-product otoacoustic emissions testing

DPOAEs were measured bilaterally before and after treatment in nine patients (Fig. 1C and D). DPOAE amplitudes shifted, on average, by −0.62 dB ± 0.49 (range: −15.4 to 16.4 dB) in the right ear and by −1.32 dB ± 0.56 (range: −27 to 8.1 dB) in the left ear. Four patients (44%) demonstrated shifts at two consecutive frequencies that extended beyond the reference limits outlined by Reavis et al. (2015)^19^. Three of these patients experienced negative or reduced DPOAE amplitudes relative to pre-treatment (Pt 7, both ears; Pt 8 and Pt 11, left ear only), whereas one patient showed positive or increased DPOAE amplitudes (Pt 4, left ear only). The positive and negative variations in amplitude were not consistent across the frequency range assessed. Although we did not observe significant changes in audiometric thresholds in this cohort, 30% of patients in this study demonstrated subtle changes in DPOAEs that are considered outside the normal reference limits. These data are consistent with possible sub-clinical changes in the function of cochlear outer hair cells.

### Speech audiometry

Word recognition scores (WRS) in quiet were available pre-and post-treatment for eight patients (Fig. 1E). WRS percentages were typically classified as follows: Excellent (90–100%), Good (75–89%), Fair (60–74%), Poor (50–59%), or Very Poor (below 50%)^20^. In our study, 12 ears (75%) had Excellent baseline WRS while 4 (25%) had Good baseline WRS. At follow-up, 14 ears (88%) had Excellent WRS while two (12%) had Good. On average, WRS performance improved relative to baseline by 3.1% ± 2% (Range: −24% to + 8%) which is within the expected test-retest range given the use of a 25-word list^21^. The Wilcoxon matched-pairs signed rank test showed that there was no significant difference in WRS for the right (*p* = 0.4688) and left (*p* = 0.5000) ears (Fig. 1C). Thus, we did not observe any significant changes in word recognition scores post-ICB therapy.

## Discussion

Ototoxic risk of some systemic treatments for cancer, such as platinum-based chemotherapy, is well established; however, this risk is poorly understood for patients receiving ICB^6^. Previous studies assessing ototoxicity risk with ICB treatment have been small retrospective case series of heavily pre-treated patients, many of whom did not undergo baseline audiometric assessments. The lack of baseline testing in these studies makes it impossible to determine if otologic dysfunction is attributable to ICB treatment^8,22^. Here, we report the results of audiologic assessment before and after neoadjuvant ICB in a small cohort of 12 patients. We did not observe clinically meaningful changes in hearing function in our cohort.

The observed shifts in DPOAEs may be suggestive of potential sub-clinical changes in outer hair cell function that did not result in measurable changes in audiometric thresholds or word recognition scores. DPOAEs are reflective of outer hair cell function and are sensitive to changes that can precede changes in hearing thresholds. Our observation of DPOAE amplitude shifts that are outside of the normal test-retest range at two or more consecutive frequencies in over half of treated patients likely reflect changes in outer hair cell function that are not sufficient to result in a change in hearing sensitivity^19^. However, it is important to acknowledge that DPOAE results can be influenced by technical factors such as consistency in probe placement and middle ear status, which may limit the interpretability of some individual findings. Overall, the clinical significance of these amplitude changes in our study is unclear, but they suggest that long-term monitoring of otologic function should be considered in patients who receive ICB treatment.

There are reports of hearing loss in patients after receiving cancer immunotherapy treatment, with the development of hearing and/or balance dysfunction as early as two weeks after initiation of treatment^9^. Given that the hypothesized mechanisms of ICB-associated ototoxicity include the development or expansion of adaptive T-cell responses against antigens expressed in cells within the inner ear, ICB dose and duration of treatment may influence the risk of ototoxicity^10,23^. Although our results are consistent with a low risk of acute ototoxicity from neoadjuvant ICB, it is possible that long-term follow-up of such patients could reveal the emergence of late ototoxicity.

### Limitations

Although this is the largest reported dataset study to date measuring audiologic function before and after ICB treatment, it is a small sample size, and the short duration of treatment and narrow audiometric testing window are additional limitations that preclude comprehensive examination of the risk of ototoxicity. ASHA and CTCAE criteria are widely used to detect ototoxicity and were appropriately applied in this study, but it is important to note that these guidelines primary focus on changes in auditory thresholds and may not capture subclinical cochlear changes or functional hearing deficits that may be better detected using speech-in-noise testing. Future monitoring protocols may benefit from inclusion of expanded metrics to better characterize the safety profile of ICB treatment. Additionally, evidence of fluctuations in DPOAE amplitude which suggest subtle changes in cochlear function should be interpreted with caution given that the reported reference limits extend only to 15 days from baseline.

## Conclusion

This retrospective study did not find evidence of acute clinically significant changes in hearing in a small cohort of individuals with newly diagnosed HNSCC treated with neoadjuvant ICB. However, prospective, long-term monitoring for potential ototoxicity in patients who receive any duration of ICB treatment could provide more insight regarding the early DPOAE changes that we observed. Additionally, the use of extended high-frequency audiometric testing may be a more sensitive approach to characterize early ototoxicity-associated hearing loss in patients receiving ICB treatment^24^.

## Data Availability

Data will be made available upon request. Contact Katharine Fernandez at katharine.fernandez@nih.gov.
